# Identification of the Volatile Compounds and Sensory Attributes of Long-Term Aging Vin Santo Wine from Malvasia di Candia Aromatic Grapes

**DOI:** 10.3390/foods9121736

**Published:** 2020-11-25

**Authors:** Monica Laureati, Camilla Cattaneo, Fernando Tateo, Monica Bononi

**Affiliations:** 1 Department of Food, Environmental and Nutritional Sciences (DeFENS), University of Milan, 20133 Milan, Italy; monica.laureati@unimi.it; 2 Department of Agricultural and Environmental Sciences (Di.S.A.A), University of Milan, 20133 Milan, Italy; fernando.tateo@unimi.it (F.T.); monica.bononi@unimi.it (M.B.)

**Keywords:** passito wines, sensory profiling, aroma compounds, aged Vin Santo wines

## Abstract

In an effort to offer a contribution to fill the gap of knowledge about the relationship between the sensory properties and aromatic profile of Malvasia grapes, the present work was aimed at evaluating volatile compounds, aroma, and sensory attributes of long-term aging (15 years) Vin Santo wine obtained from Malvasia di Candia aromatica grapes. In this article, the aromatic profile are studied using gas chromatography-mass spectrometry (GC-MS), gas chromatography with flame ionization detection (GC-FID), and sensory analysis by involving a panel of trained assessors to explore the sensory profile resulting after long-term aging (up to 15 years). The GC-MS and GC-FID analyses of wines allowed the identification and semi-quantification of twenty-seven volatiles belonging to 12 conventional groups of compounds. From a sensory perspective, the Vin Santo wines analyzed in this study presented a very complex sensory profile characterized by 19 sensory descriptors of which 14 related to olfactory terms. The relationship between sensory and GC-FID data deduced from three samples representing nearly three years in the past 15 years was investigated by means of Partial Least Square (PLS) modeling, showing that specific volatile compounds could predict a specific orthonasal and/or retronasal odor perceived by the trained panel of assessors, clearly differentiating the Vin Santo vintages. Identifying the main volatiles and aromas of long-term Vin Santo wine may be helpful to winemakers, since wine aging sensory properties are often associated with a prestigious image and contribute to defining wine quality.

## 1. Introduction

Vin Santo is a traditional term referring to a variety of dessert wines that have been historically produced in Italy and Greece. In Italy, Vin Santo wines are commonly named Passito wines, which literally means wines produced from dried grapes [1]. The major production areas are settled, particularly, in Tuscany and in some other northern and central viticulture areas of Italy [2]. Today, Italian Vin Santo is recognized and protected by EU regulations as a Quality Wine Produced in Specific Regions (QWPSR) under the specific Protected Designation of Origin (PDO). The European legislation [3] defines “Vin Santo” as follows:

“Vin Santo”, “Vino Santo”, “Vinsanto” is a historical-traditional term related to some wines produced in Italian regions such as Toscana, Marche, Umbria, Emilia Romagna, Veneto, and Trentino Alto Adige. It corresponds to a particular wine typology and to the corresponding complex production method which implies storage and wine grapes drying in suitable and aerated places for a long aging period into traditional wooden containers.

Italian Vin Santo wines traditionally follow the same production method, although there are major differences in the must composition (grape varieties and grape drying), fermentation, and aging conditions, which lead to a great diversity of Vin Santo sensory quality [1]. Vin Santo is made by choosing the best grapes (‘scelti’, literally selected) of the white varieties that grow in a specific production area. A blend of non-varietal grapes (i.e., grapes not belonging to a single production area, e.g., Trebbiano) and aromatic (e.g., Malvasia) or semi-aromatic grapes (e.g., Grechetto) is made to confer to the final product the typical aromatic style. After harvesting, grapes are partially dried indoors under ambient conditions or directly on the sun for a period long enough (usually 3–4 months) to achieve a sugar concentration ranging from 26% to 48%. After having separated the healthy berries from the rot and damaged ones, dried grapes are pressed, resulting in a high-sugar-containing must, which is left to settle for 3–4 days. Then, the decanted juice is fermented and matured in 50 L to 200 L traditional wooden barrels (usually chestnut, oak, or cherry) known as ‘caratelli’ for a period of 2–4 or even more years in the cellar or ‘vinsantaia’, which is a traditional room usually located in the attic of wineries, with the ventilation managed by opening the windows, thus being more subjected to seasonal temperature variations [1,4]. According to the traditional process, during maturation, the wines are subject to fluctuation in seasonal temperatures [2] which can be extremes, especially if ‘vinsantaia’ is chosen, and in relation to the length of the aging period and to the microbial population biodiversity involved in the fermentation [5,6]. Significant variations in the chemical and physical wine composition occur with important consequences, not always foreseeable, on perceived sensory properties [1].

The different types of Vin Santo are characterized by complex sensory profiles, which mainly arise from the blend of grapes and the winemaking process used. In general, this winemaking process provides demi-sec style (10–50 g/L of residual sugar content) or slightly sweet or sweet style wines (up to 100 g/L of residual sugar content) characterized by pale to dark amber color and by raisin, nutty, honey and hay notes, with generally high alcohol content (14% and above) [7,8]. Moreover, Vin Santo body can vary from low structure to full-body depending on the net extract content [1].

To the best of our knowledge, there are no published data on Colli Piacentini DOC (Denominazione di Origine Controllata) Vin Santo, and few studies have been carried out on Malvasia grapes considering its sensory properties and relating them to the aromatic profile. The wines evaluated in the present study were produced by Malvasia di Candia aromatica, a grapevine (*Vitis vinifera L.*) cultivar (cv.) that produces aromatic white grapes, cultivated in Emilia Romagna in the area of Reggio Emilia, Parma, and Piacenza, while in Lombardy it is cultivated in the area of Oltrepò Pavese. This cv. is used to produce semi-sparkling, semi-dry or sweet wines, and more recently, dry or passito wines [9].

Although Vin Santo is a niche product, its production has increased over the years, following the general increase in demand for sweet wines, corresponding to +20% [1,10]. Due to the great influence that winemaking process and grape variety may have on the quality properties of Vin Santo wine, and due to the low standardization of Vin Santo winemaking, so far, there have been few scientific studies that have focused on long-term Vin Santo aging [1,11].

Wine aging starts at the end of fermentation and continues after bottling until consumption. Its duration is variable depending on the wine’s origin, type, and quality. Many changes occur in wine composition during this period, including the development of color, aroma, and flavor [12]. The speed of such a transformation is not the same for all wines as it depends on their initial composition and cellar conditions [13]. Chemical reactions of the phenolic fraction, resulting in the formation of oligomeric and polymeric derivatives, are the major contributors to wine aging [12,13]. With aging, the color gradually changes from cherry-red to deep red, and then brick-red for red wines; while for white wines, color changes from pale yellow to amber, deep-yellow-orange [12]. Concerning odor, development of wine’s aromatic profile during aging usually includes the loss of fermentative aromas, a variable attenuation of fresh fruity note, retention of varietal aromas, and an evolution towards more complex and subtle aromas, resulting in a homogeneous, harmonious flavor [14]. During aging, the wine’s aromatic profile develops from primary and secondary aromas or metabolites, under the influence of outside parameters, including aging in wooden barrels and oxygen levels during bottle storage [15,16,17]. Oral sensations, such as astringency and body, are also subject to modification during aging, as tannins undergo transformations that produce a reduction in astringency perception [12]. Although mainly associated with red wines, aging also applies to white wines. Recently, the effect of aging on white wines has been evaluated in a number of studies [18,19,20,21,22,23,24,25,26,27,28], but little attention has been given to Vin Santo wine. Domizio et al. [2] correlated the complex phenomena during maturation with the chemical and sensory characteristics of Vin Santo made under different experimental conditions (i.e., different inoculum of starter strains), although the data reported are referred to a relatively short aging period of 18 months. Moreover, very few papers have been published that deals with identifying the volatile compounds and sensory properties of long-term aging.

In this regard, the present study was carried out to make a contribution to the knowledge of the resulting sensory properties that characterize Vin Santo long-term aging (up to 15 years) by analyzing its aromatic profile through GC-MS, GC-FID, and sensory characteristics involving a panel of trained assessors. To carry out a preliminary study on the sensory effect of aging of Vin Santo produced by Malvasia di Candia aromatic grapes, the samples available at the producer and derived from 3 vintages (2004, 2005, 2006) were examined. These samples represent today the average result of the evolution of the sensory properties correlated to analytical data detected after 15 years of aging. Identifying the main volatiles and sensory properties of long-term Vin Santo wine may be helpful to winemakers, since the wine aging bouquet is often associated with a prestigious image, linked to tradition and refined winemaking, and clearly contributes to defining wine quality [14].

## 2. Materials and Methods

### 2.1. Chemicals and Reagents

Ethanol (≥99.8%) and water for high-performance liquid chromatography (HPLC) was purchased from Carlo Erba Reagents (Milan, Italy), while ethyl octanoate (≥98%) and sodium sulfate anhydrous (≥99.0 %) were purchased from Sigma Aldrich (Milan, Italy).

### 2.2. Standard Solutions

For semi-quantitative analysis, a stock solution of ethyl octanoate was prepared by dissolving the compound in 10% (*v*/*v*) ethanol solution. Working standard solutions were prepared to cover two range of concentration: 0.2–2.0–20.0–40.0–100.0–200.0 µg L^−1^ (R^2^ = 0.994) and 200.0–400.0–1000.0–2000.0 µg L^−1^ (R^2^ = 0.998). These two calibration curves were used for estimating the order of magnitude of the 27 compounds semi-quantified by GC-FID and identified by GC/MS (minimum percentage of threshold reliability 95% by National Institute of Standards and Technology (NIST) 147 library spectra database and 97% by DIFCA–UniMi 2017 (today “Di.S.A.A. library”) flavor spectra specific database; data expressed as µg L^−1^ of ethyl octanoate). “Di.S.A.A. library” was made initially with standard certified compounds and also with some commercial compounds. Over the years, all the commercial compounds were confirmed by standard certified compounds.

### 2.3. Wine Samples

The wine analyzed in the present study was a Colli Piacentini DOC Vinsanto Albarola Val di Nure produced and bottled by a wine company from the area of Piacenza (Italy). Colli Piacentini DOC Vinsanto di Albarola is a Vin Santo exclusively made from Malvasia di Candia aromatic grapes. Three vintages (2004, 2005, and 2006) were evaluated in duplicate for GC-MS, GC-FID, and sensory parameters in spring 2019. All the samples considered were at about 10% (*v*/*v*) alcoholic degree.

### 2.4. Sample Preparation for GC-FID and GC-MS Analysis

To 20 mL of each wine were added 2 mL of ethyl alcohol and accurately shacked. Small quantities of sodium sulfate anhydrous were added gradually, and were mixed with care. The resulting residue alcoholic fraction was isolated from the crystallized sodium sulfate by centrifugation at 5000 rpm, and 2 µL of the supernatant were injected directly in GC-MS and in GC-FID.

Ethyl alcohol was added to allow easier recovery of the liquid fraction containing the volatile compounds. A semi-quantitative analysis considered the resulting dilution of the alcoholic fraction of the matrix, and the total water absorbed from the system by sodium sulfate anhydrous.

### 2.5. GC-MS Analysis

The GC-MS analyses were carried out using a Shimadzu 2010 gas chromatograph coupled to a Shimadzu QP-2010 MSD quadrupole mass spectrometer (Shimadzu, Milan, Italy). A Restek Rxi-5ms 30 m × 0.25 mm, 0.25 µm film thickness capillary silica column (Restek, Milan, Italy) was used for the volatile compounds’ separation. The operating conditions were: Helium flow 1.0 mL min^−1^ and oven temperature 40 °C for 1 min, increased to 60 °C at a rate of 2 °C min^−1^, increased to 240 °C at a rate of 3 °C min^−1^, and 30 min hold; injection was in split mode (1:5), and the injector and detector temperatures were set at 220 °C and 240 °C, respectively. The MS ran in electron impact (EI) mode was at 70 eV electron energy, and the temperature of the ion source was 200 °C. Mass spectra were acquired over the mass range 40–300 a.m.u. (atomic mass unit). Volatile compounds were identified by matching their mass spectra with the reference mass spectra of an in-house databank “Di.S.A.A. library” (threshold of probability > 97%) and that of NIST 147 library (threshold of probability > 95%). The list of the volatile compounds identified is reported in Table 1.

### 2.6. GC-FID Analysis

GC-FID analyses were carried out with a Shimadzu 2010 Plus gas chromatograph (Shimadzu, Milan, Italy). Hydrogen was used as the carrier gas at a flow rate of 1.5 mL min^−1^. All the compounds were quantified using an Equity-5 capillary column poly (5% diphenyl/95% dimethyl siloxane) 60 m × 0.25 mm, 0.25 µm i.d. film thickness) (Supelco, Milan, Italy). The oven temperature program was 40 °C for 1 min, increased to 60 °C at a rate of 2 °C min^−1^, increased to 240 °C at a rate of 3 °C min^−1^, and 30 min hold 240. The injector temperature was 220 °C, and the split injector mode (1:5) was used. The detector temperature (FID) was 240 °C.

The capillary columns adopted for GC-MS and for GC-FID analyses, both with low polarity, are absolutely equivalent in resolution sequencing because they are characterized by the same stationary phase 5% poly dimethyl siloxane (i.d. 0.25 mm, film thickness 0.25 µm). Therefore, the correct identification of peaks is guaranteed.

### 2.7. Sensory Analysis

The sensory profiling method was applied to identify and quantify wines’ sensory properties [29]. Nine subjects (five women and four men aged between 20 and 60) were selected from a pool of expert panelists [30]. The method consisted of an initial training phase to acquire familiarity with the product and the methodology, followed by a second phase focused on wines evaluation. Subjects were informed about the aim of the study (i.e., analyzing the sensory profile of Vin Santo wine) and were involved in six 1 h common sessions and four sessions in individual sensory booths. During the training phase, commercial Vin Santo wines covering a wide range of variability were selected and presented to the assessors to stimulate the generation of descriptors. As training progressed, descriptive terms were defined through a panel discussion, and relevant reference standards, corresponding to the maximum intensity of the rating scale, were developed (Table 2). No further reference standards for intermediate scale intensity were used.

After the training phase, judges evaluated the three Vin Santo wines in two replicates on different days. Judges were instructed to drink and swallow each sample and rate the intensity of each attribute using a scale ranging from the minimum intensity of the sensation (score = 1) and maximum intensity of the sensation (score = 9, represented by the reference standards see Table 2). The training and evaluation sessions were performed, respectively, in the room for collective discussions and in individual sensory booths at the sensory laboratory of the Department of Food, Environmental and Nutritional Sciences (DeFENS, Università degli Studi di Milano) designed in accordance with ISO guidelines [31].

Data acquisition was done using Fizz v2.31 software (Biosystèmes, Couternon, France). Assessors were asked not to smoke, eat or drink anything, except water, at least one hour before the tasting sessions. For both the training and evaluation sessions, for each sample, judges received a 20 mL sample served in transparent ISO glasses [32] coded with a 3-digit number and covered with a Petri dish to avoid the escape of volatile components. Participants were provided with mineral water and unsalted crackers to clean their mouth between tastings. Wines were served at room temperature, which was set at 20 °C. Presentation orders were systematically varied over assessors and replicated to balance the effects of serving order and carryover [33].

### 2.8. Data Analysis

Sensory data were analyzed by means of 3-way ANOVA considering Wines (vintages 2004, 2005, 2006), judges (9 assessors), replicates (rep 1, rep 2), and their 2-way interactions as factors [29].

When the ANOVAs showed a significant effect (*p* < 0.05) for the factor Wines, the Least Significant Difference (LSD) was applied as a multiple comparison test.

The relationship between the sensory attributes (odors and flavors) and the GC-FID data was studied by means of the Partial Least Square (PLS) regression [34]. PLS procedure models both the X- and Y- matrices simultaneously to find the variables in X that will best predict the variables in Y. These PLS components are similar to principal components from principal component analysis (PCA), but will be referred to as factors or latent variables or latent structures. In PLS models, Scores and Loadings express how the samples and variables are projected along with the model factors. In this experiment, sensory attributes related to orthonasal (odor) and retronasal (flavor) perception, averaged across judges, were used as Y matrix, and GC-FID data were set as X matrix. Autoscaling was performed on the data prior to any modeling. This pre-processing technique is required when variables showing different variation ranges need to be compared. It gives all variables the same chance to influence the estimation of the components. Cross-validation was chosen as the validation method. A preliminary PLS model was run considering all variables (all volatile compounds, as well as all orthonasal and retronasal sensory descriptors). A correlation loadings plot was used to find variables with less than 50% explained variance, which were left out of the model [35]. This resulted only in the omission of one volatile compound, i.e., ocimene. Variable importance for the projection (VIP) was calculated. VIP values make it possible to assess the importance of the variables for prediction in a model [36]. According to Tenenhaus et al. [36], the variables are regarded important for the prediction if VIP scores are higher than 0.8.

Data were treated using SAS/STAT statistical software package version 9.1.3. (SAS Institute Inc., Cary, NC, USA) and XLSTAT (version 2019.2.2, Addinsoft, Boston, MA, USA).

## 3. Results and Discussion

### 3.1. GC-MS and GC-FID Analysis

All analyses were performed in duplicate, and the results of GC-FID analysis are reported in Table 3 expressed as µg L^−1^ of ethyl octanoate (mean values). The GC-MS analysis of wines identified twenty-seven compounds belonging to 12 groups of volatile compounds. The volatile compounds responsible for the aroma were identified by GC-MS and by comparison with GC-FID on alcoholic extracts produced from wines.

In this work, we have identified three monoterpenes, three alcohols, one C_6_-compounds, ten ethyl esters, one volatile fatty acid, two acetates, and seven other compounds (i.e., aldehydes, heterocycle compounds, other esters, etc.). Many of these volatile compounds, which derive from grapes cultivar, yeast strain fermentation, and vinification process, are commonly found in wines. The aromatic profile of Malvasia di Candia aromatica cultivar has been previously studied in relation to cultivation techniques, terroir, as well as to winemaking techniques [37]. Masino et al., [38] studied various accessions of Malvasia di Candia aromatica collected in the area around Reggio Emilia grapes. Terpenoid profile was particularly rich and varied, being recognized as compounds responsible for the typical varietal aroma [37,39,40]. The total concentration of free monoterpenes, generally, allows the distinction between aromatic and non-aromatic grapevine varieties [41].

In agreement with previous research [9,42,43], in the present study, we identified some terpenic alcohols and derivatives, such as alpha terpineol, limetol, and linalool oxide. The free forms of monoterpenes are normally degraded both during drying, maturation, and alcoholic fermentation thanks to the action of yeast. The concentration of this fraction is further reduced during wine-aging, through a chemical transformation that leads to the formation of other volatile compounds [43]. Higher alcohols and esters, produced during alcoholic fermentation, play an important role in the flavor of wines, depending on the type of compound and concentration [43]. Amyl and isoamyl alcohols, are reported to increase regularly during the winemaking process [43], while phenethyl alcohol derives from both varietal characteristic and fermentative process [44]. Moreover, the C_6_-alcohol has a pre-fermentative origin, but could also be involved in yeast metabolism [44]. The volatile fatty acids and their ethyl esters are fermentation compounds that deserve special attention for their sensory characteristics, related to both wine’s pleasant note and peculiarity [43,45,46,47].

### 3.2. Sensory Analysis

Nineteen sensory descriptors covering appearance (amber color), odor (solvent, honey, caramel, rum, dried figs, baked apple, nutty), taste (sweet and sour), flavor (solvent, honey, caramel, rum, dried figs, baked apple, nutty) and mouthfeel (body, alcohol) were defined.

Mean intensity ratings of Vin Santo wines are reported in Table 4. ANOVA results showed that all descriptors, except nutty flavor, discriminated significantly against the wines in the three vintages.

In general, the Vin Santo wines analyzed in the present study were highly aromatic, with 14 olfactory descriptors detected by the trained panel. Accordingly, in a study by Mazzaglia et al. [48], Malvasia wines from different Mediterranean areas were evaluated for their sensory properties, and in agreement with the present study, found a very complex sensory profile characterized by 21 sensory descriptors of which 17 related to olfactory terms. The samples evaluated in the present study, had a high sweet taste intensity and were full-bodied but, differently from traditional Vin Santo wines, they were not as much as high in alcohol perception. The total acidity was not negligible, sour perception by the sensory panel was reduced, probably due to the high intensity of sweet taste, which is known to modulate the perception of acid compounds [49]. Differently from previous studies [1,2,48], Colli Piacentini DOC Vin Santo wines were not perceived as astringent. This might be due to the high intensity of sweet taste and body that can modulate the perception of bitter and astringent compounds, respectively [50,51]. Vintage 2004 was also perceived as darker than the other wines. It is well known that during maturation, oxygen promotes deep changes in the phenolic compounds. In particular, polyphenol oxidation determines the production of quinone, and their polymerization results in yellow-brown compounds responsible for wine browning reactions as aging proceeds [52,53,54]. As a consequence, Vin Santo can be characterized by a natural color that ranges from golden straw to intense amber [1].

Partial least square (PLS) regression and variable importance in the projection (VIP) were used to study the relationship between sensory data and GC-FID data, and to establish which volatile compound could predict a specific orthonasal and/or retronasal odor perceived by the trained panel of assessors.

**Figure 1:**
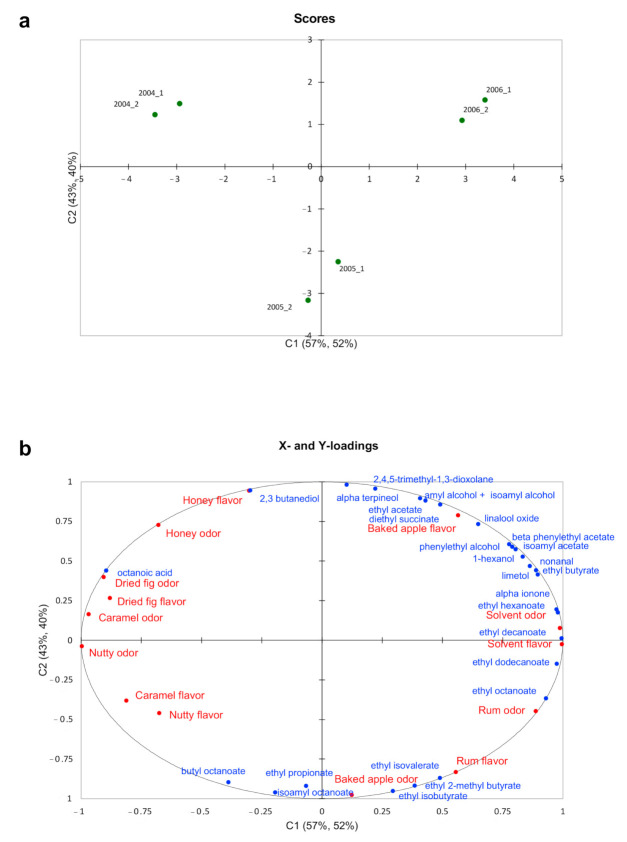
Scores (**a**) and Loadings (**b**) plots obtained by PLS modeling carried out on Vin Santo sensory aroma profile data (Y) and volatile compounds data by GC-FID (X). C1: first factor; C2: second factor.

The positioning of the Vin Santo vintages in the two replicates is reported in the Scores plot (Figure 1a), whereas the relation between sensory data (red font) and volatile compounds (blue font) is reported in the Loadings plot (Figure 1b).

The first factor (C1) explains, respectively, the 57% and 52% of the variation in Y, while the second factor (C2) accounts for, respectively, the 43% and 40%. The cumulative Q^2^ of the model was 0.95%, reflecting an excellent relationship between the sensory and GC-FID data, while the cumulated R^2^Y and R^2^X cum corresponding to the correlations between the explanatory (X) and dependent (Y) variables with the components are very close to 1 (R^2^Y = 0.97 and R^2^X = 0.94, respectively).

The first factor clearly differentiated the 2004 vintage (on the upper left pane) from the 2006 vintage (on the upper right pane), whereas the second factor differentiated vintage 2005 (on the lower panes) from the other wines (Figure 1a). Looking at Figure 1b, it is possible to gain information about the fingerprint of these three vintages. Vintage 2004 was mainly correlated to the sensory attributes honey, dried fig, caramel, and nutty odors and flavors, which are usually associated with oxidation notes [55]. These aroma sensory descriptors were related to octanoic acid and 2,3 butanediol, which have been reported to be responsible for sweetie and fatty notes [56]. On the contrary, vintage 2004 had the lowest perceived intensity of solvent and rum odors and flavors, which were the main descriptors characterizing vintages 2006 and 2005, respectively. These sensory descriptors were associated with volatile compounds, such as ethyl dodecanoate, and ethyl and isoamyl octanoate—responsible for alcoholic (cognac/brandy) notes—as well as ethyl proprionate and ethyl isobutyrate—responsible for ethereal, alcoholic, fusel, and pungent notes [55]. Vintages 2005 and 2006 were also described by the sensory descriptors baked apple odor (2005) and flavor (2006). Coherently, these vintages were also associated with higher concentrations of ethyl isovalerate and ethyl hexanoate (apple odor), as well as isoamyl acetate and diethyl succinate (apple flavor), which have been reported to be responsible for the perception of a fruity-apple odor [43,54,56,57,58].

**Figure 2:**
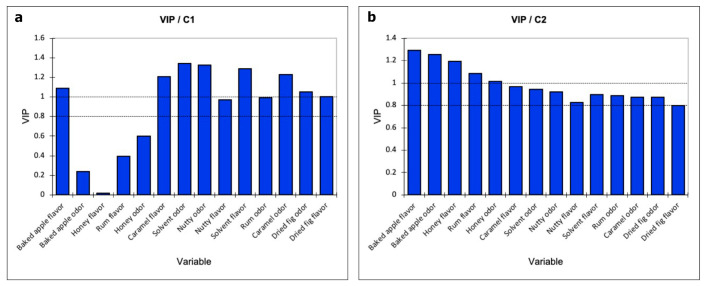
Variable importance in projection (VIP) values for the partial least squares analysis. (**a**) VIP indices on C1; (**b**) VIP indices on C2.

On the basis of the data obtained by PLS modeling, variable importance in projection (VIP) was used to extract the most relevant volatile compounds for each sensory attribute (See supplementary Table S1). VIP results from a weighted sum of squares of the PLS-loadings, and it is related to the amount of explained Y-variable in each dimension. Figure 2 reported the VIP indices associated with a PLS model comprised of the first two components, allowing the identification of the explanatory variables that contribute the most to the model. The analysis showed that the baked apple odor, honey flavor, and odor, and rum flavor have low VIP indices (smaller than 0.8) on C1 (Figure 2a). Nevertheless, looking at the VIP indices on the second component all the variables show a great influence (Figure 2b).

All together, these results allow the definition of the sensory characteristics of Vin Santo wine aged up to 15 years and consider for the first time the opportunity of pushing so far the aging process in Vin Santo from Malvasia di Candia grapes. Nor off-odors neither off-flavors have been identified in the samples, suggesting that the 15-year aging limit can still be considered acceptable for the quality of wines. However, further studies, are needed to investigate this aspect, especially taking into consideration the consumers’ point of view regarding sensory acceptability.

## 4. Conclusions

In the present work, the characterization of volatile compounds and sensory attributes of long-term aging Vin Santo wine obtained from Malvasia di Candia aromatica were evaluated for the first time. Volatile compounds were identified by GC-MS on alcoholic extracts of wines, and a semi-quantitative analysis was carried out by GC. It was possible to identify 27 compounds belonging to 12 groups of volatiles, and a sufficient measure of priority was identified among the molecules responsible for the aroma.

From a sensory point of view, the Vin Santo wine analyzed presented a very complex sensory profile characterized by 19 sensory descriptors of which 14 related to olfactory terms, especially characterized by sweetie and fatty notes, solvent and rum odors and flavors, as well as apple odor and flavor. The relationship between sensory and analytical data investigated by means of the PLS modeling showed that specific volatile compounds could predict a specific orthonasal and/ or retro nasal odor perceived by the trained panel of assessors.

The combined approach can be useful to producers to characterize the sensory quality of wines during aging to define the most acceptable aging limit, given that the development of the aroma was also evident within three years. Finally, it is important to note that, in the present work, due to the selection of a limited number of vintages of only one producer, the generalization of the results was not possible. Therefore, future studies should consider different vintages, including younger ones, to define the evolution of aromatic profile over time.

## Figures and Tables

**Table 1:** Volatile compounds identified by GC-MS.

Compound
ethyl acetate
ethyl propionate
2,4,5-trimethyl-1,3-dioxolane
amyl alcohol + isoamyl alcohol
ethyl isobutyrate
2,3 butanediol
ethyl butyrate
ethyl 2-methyl butyrate
ethyl isovalerate
1-hexanol
isoamyl acetate
limetol
ethyl hexanoate
ocimene quintoxide
linalool oxide
nonanal
phenethyl alcohol
diethyl succinate
alpha terpineol
octanoic acid
ethyl octanoate
β-phenethyl acetate
α-ionone
butyl octanoate
ethyl decanoate
isoamyl octanoate
ethyl dodecanoate

**Table 2:** Sensory vocabulary used by the trained panel of judges to describe the sensory profile of Vin Santo wines during aging with definition and reference standards for each term elicited. If not differently reported, the standard reference refers to the maximum intensity of the scale.

Sensory Descriptor	Definition	Reference Standard
APPEARANCE		
Amber color	Characteristic perceived through the sense of sight referring to the orange-yellow color reminiscent of amber material	Rum Puerto Oscuro (CFL S.p.A., Finale Emilia, Italy)
ODOR (Orthonasal perception)		
Solvent	Characteristic perceived through the sense of smell and reminiscent of the nails polish remover	2 mL of acetone in 300 mL of Tavernello white table wine (Caviro S.p.A., Faenza, Italy)
Honey	Characteristic perceived through the sense of smell and reminiscent of chestnut honey	Infusion (24 h, 5 °C) of 50 g of chestnut honey (Apicoltori riuniti Abello, Soc. Coop. Agricola a.r.l, Asti, Italy) in 300 mL of Tavernello white table wine (Caviro S.p.A., Faenza, Italy)
Caramel	Characteristic perceived through the sense of smell and reminiscent of caramel (burnt sugar)	2 mL of caramel aroma (Funcakes Caramel Pasta aromatizzante, Newcakes, NL) in 300 mL white wine (Caviro S.p.A., Faenza, Italy)
Rum	Characteristic perceived through the sense of smell and reminiscent of alcoholic spirits (rum)	Rum Puerto Oscuro (CFL S.p.A., Finale Emilia, Italy)
Dried fruit (figs)	Characteristic perceived through the sense of smell and reminiscent of dried fruits, especially dried figs	Infusion (24 h, 5 °C) of 150 g of organic dried figs (EcorNaturaSì S.p.A, Verona, Italy) in 300 mL of Tavernello white table wine (Caviro S.p.A., Finale Emilia, Italy)
Baked fruit (apple)	Characteristic perceived through the sense of smell and reminiscent of baked fruits, especially apple	Infusion (24 h, 5 °C) of 40 g apple puree (La mousse, Chini Srl, Trento, Italy) in 300 mL of Tavernello white table wine (Caviro S.p.A., Faenza, Italy)
Nutty	Characteristic odor of nuts (walnuts and almonds) perceived through the sense of smell	Infusion (24 h, 5 °C) of 45 g of organic walnuts (Mezze noci sgusciate, Rapunzel Naturkost GmbH, Legau, Germany) and 45 g of toasted organic almonds (Mandorle sgusciate Carrefour, GS S.p.A., Milano, Italy) in 300 mL of Tavernello white table wine (Caviro S.p.A., Faenza, Italy)
TASTE		
Sweet	Basic taste elicited by sugars (e.g., table sugar) and perceived in the oral cavity	130 g of fructose in 500 mL of Tavernello white table wine (Caviro S.p.A., Faenza, Italy)
Sour	Basic taste elicited by sour compounds and perceived in the oral cavity	0.20 g of tartaric acid in 300 mL of Tavernello white table wine (Caviro S.p.A., Faenza, Italy)
FLAVOUR (retronasal odor perception)		
Solvent	Characteristic odor perceived through the senses of smell, taste, and touch perceived during swallowing and reminiscent of the nails polish remover	2 mL of acetone in 300 mL of Tavernello white table wine (Caviro S.p.A., Faenza, Italy)
Honey	Characteristic odor perceived through the senses of smell, taste, and touch perceived during swallowing and reminiscent of chestnut honey	Infusion (24 h, 5 °C) of 50 g of chestnut honey (Apicoltori riuniti Abello, Soc. Coop. Agricola a.r.l, Asti, Italy) in 300 mL of Tavernello white table wine (Caviro S.p.A., Faenza, Italy)
Caramel	Characteristic odor perceived through the senses of smell, taste, and touch perceived during swallowing and reminiscent of caramel (burnt sugar)	2 mL of caramel aroma (Funcakes Caramel Pasta aromatizzante, Newcakes, NL) in 300 mL white wine (Caviro S.p.A., Faenza, Italy)
Rum	Characteristic odor perceived through the senses of smell, taste, and touch perceived during swallowing and reminiscent of alcoholic spirits (rum)	Rum Puerto Oscuro (CFL S.p.A., Finale Emilia, Italy)
Dried fruit (Figs)	Characteristic odor perceived through the senses of smell, taste, and touch perceived during swallowing and reminiscent of dried fruits, especially dried figs	Infusion (24 h, 5 °C) of 150 g of organic dried figs (EcorNaturaSì S.p.A, Verona, Italy) in 300 mL of Tavernello white table wine (Caviro S.p.A., Faenza, Italy)
Baked fruit (apple)	Characteristic odor perceived through the senses of smell, taste, and touch perceived during swallowing and reminiscent of baked fruits, especially apple	Infusion (24 h, 5 °C) of 40 g of apple puree (La mousse, Chini Srl, Trento, Italy) in 300 mL of Tavernello white table wine (Caviro S.p.A., Faenza, Italy)
Nutty	Characteristic odor of nuts (walnuts and almonds) perceived through the sense of smell, taste, and touch during swallowing	Infusion (24 h, 5 °C) of 45 g of organic walnuts (Mezze noci sgusciate, Rapunzel Naturkost GmbH, Legau, Germany) and 45 g of toasted organic almonds (Mandorle sgusciate Carrefour, GS S.p.A., Milano, Italy) in 300 mL of Tavernello white table wine (Caviro S.p.A., Faenza, Italy)
MOUTHFEEL SENSATIONS		
Body	Characteristic perceived in the oral cavity, due to the friction among the molecules in a liquid, that gives to it a limited fluidity and mobility	30 mL of glycerol in 300 mL of Tavernello white table wine (Caviro S.p.A., Faenza, Italy)
Alcohol	Characteristic heat/burning sensation perceived in the oral cavity	45 mL of 95% ethyl alcohol (Alcol Buongusto, Carrefour, Milano, Italy) in 300 mL of Tavernello white table wine (Caviro S.p.A., Faenza, Italy)

**Table 3:** Volatile compounds composition in the three vintages. Data are expressed as µg L^−1^ of ethyl octanoate (SD = standard deviation).

Compound	Vintage					
	2004		2005		2006	
	Mean	SD	Mean	SD	Mean	SD
**Terpenoids**						
Alpha terpineol	26.53	0.14	7.72	0.20	29.1	1.63
Limetol	52.97	0.82	64	0.30	174.27	85.59
Linalool oxide	13.78	0.04	11.34	0.07	18.81	3.72
**Sum**	93.28		83.06		222.18	
**Alcohols**						
Amyl alcohol + isoamyl alcohol	323.37	5.78	279.61	1.53	366.64	30.21
2,3-butanediol	18.56	0.06	2.34	0.08	13.72	3.52
**Sum**	341.93		281.95		380.36	
**Aromatic alcohols**						
Phenethyl alcohol	106.93	2.28	96.96	0.14	172.02	46.08
**C6-compounds**						
1-hexanol	13.73	0.30	12.45	0.16	24.09	7.27
**Volatile fatty acids**						
Octanoic acid	19.55	0.08	1.41	0.04	0	13.82
**Ethyl esters**						
Ethyl acetate	29.55	0.10	11.67	0.18	42.4	8.78
Ethyl butyrate	7.57	0.07	7.72	0.06	9.7	1.34
Ethyl decanoate	18.93	0.08	203.87	0.44	401.81	270.54
Ethyl dodecanoate	4.6	0.03	8.41	0.28	10.71	4.70
Ethyl hexanoate	112.12	2.60	169.6	0.54	291.94	127.39
Ethyl isobutyrate	7.8	0.31	35.23	0.04	18.19	7.25
Ethyl isovalerate	6.47	0.03	37.15	0.14	15.48	6.49
Ethyl 2-methyl butyrate	4.4	0.11	14.27	0.07	9.18	3.45
Ethyl octanoate	263.29	1.30	1350.81	7.69	1560.02	915.96
Ethyl propionate	16.38	0.06	19.91	0.08	15.38	0.78
**Sum**	471.11		1858.64		2374.81	
**Other esters**						
Butyl octanoate	11.09	0.10	17.73	0.31	6.53	3.34
Diethyl succinate	326.06	1.40	218.37	0.04	411.1	59.76
Isoamyl octanoate	0.63	0.01	1.03	0.08	0.58	0.08
**Sum**	337.78		237.13		418.21	
**Acetates**						
Beta-phenethyl acetate	10.21	0.24	8.67	0.11	18.81	5.94
Isoamyl acetate	36.06	0.21	34.78	0.04	59.05	16.05
**Sum**	46.27		43.45		77.86	
**Aldehydes**						
Nonanal	4.21	0.11	4.30	0.13	8.40	3.10
**Chetons**						
Alpha-ionone	0.41	0.06	2.21	0.03	5.69	3.69
**Others**						
2,4,5-trimethyl-1,3-dioxolane	71.28	0.99	37.74	0.72	81.98	7.35
Ocimene quintoxide	6.13	0.17	4.45	0.11	9.14	2.16

**Table 4:** Influence of vintage (expressed by F-values) on mean values of each sensory descriptor. Superscripts by row indicate significantly different means according to LSD post-hoc test (*p* < 0.05) (** *p* < 0.01; *** *p* < 0.001; n.s. not significant).

Sensory Descriptor	F-Values	Vintage		
		2004	2005	2006
Appearance				
Amber color	78.98 ***	7.5 ^c^	5.1 ^a^	6.2 ^b^
ODOR (Orthonasal perception)				
Solvent odor	79.38 ***	5.2 ^a^	6.3 ^b^	7.7 ^c^
Honey odor	386.70 ***	6.5 ^c^	2.4 ^a^	3.6 ^b^
Caramel odor	311.64 ***	5.7 ^c^	4.1 ^b^	3.2 ^a^
Rum odor	87.53 ***	4.5 ^a^	6.1 ^b^	6.2 ^b^
Dried fruit (figs) odor	62.47 ***	5.9 ^b^	4.5 ^a^	4.3 ^a^
Baked fruit (apple) odor	61.06 ***	2.8 ^a^	4.2 ^b^	2.9 ^a^
Nutty odor	90.25 ***	5.6 ^c^	4.7 ^b^	3.8 ^a^
TASTE				
Sweet taste	10.61 **	5.3 ^a^	5.6 ^b^	5.9 ^b^
Sour taste	46.07 ***	5.3 ^b^	4.5 ^a^	4.3 ^a^
FLAVOR (Retronasal perception)				
Solvent flavor	21.44 ***	2.0 ^a^	2.4 ^b^	2.7 ^c^
Honey flavor	22.20 ***	6.4 ^b^	5.4 ^a^	6.1 ^b^
Caramel flavor	11.26 ***	5.3 ^b^	5.2 ^b^	4.7 ^a^
Rum flavor	51.44 ***	4.7 ^a^	6.3 ^c^	5.6 ^b^
Dried fruit (figs) flavor	19.73 ***	6.4 ^b^	5.6 ^a^	5.5 ^a^
Baked fruit (apple) flavor	92.55 ***	3.9 ^a^	3.4 ^a^	4.6 ^b^
Nutty flavor	1.80 n.s.	3.9	3.9	3.7
MOUTHFEEL SENSATIONS				
Body	58.46 ***	6.9 ^b^	5.7 ^a^	5.7 ^a^
Alcohol	22.04 ***	5.8 ^b^	5.1 ^a^	5.2 ^a^

## Supplementary Materials

The following are available online at https://www.mdpi.com/2304-8158/9/12/1736/s1, Table S1: VIP coefficients of the models corresponding to each dependent variable.
