# Supplementary material for: Identification of the Volatile Compounds and Sensory Attributes of Long-Term Aging Vin Santo Wine from Malvasia di Candia Aromatic Grapes
Source: Foods. 2020 Nov 25;9(12):1736. doi: 10.3390/foods9121736 (PMC7759802; doi:10.3390/foods9121736)
Supplement: Supplementary file 1 [file foods-09-01736-s001.pdf]

Article

Identification of the Volatile Compounds and Sensory Attributes of Long-Term Aging Vin Santo Wine from Malvasia di Candia Aromatic Grapes

Monica Laureati [1], Camilla Cattaneo [1*], Fernando Tateo [2] and Monica Bononi [2]

1 Department of Food, Environmental and Nutritional Sciences (DeFENS), University of Milan, Via Celoria 2, 20133, Milan, Italy; monica.laureati@unimi.it (M.L.); camilla.cattaneo@unimi.it (C.C.)

2 Department of Agricultural and Environmental Sciences (Di.S.A.A), University of Milan, Via Celoria 2, 20133, Milan, Italy; fernando.tateo@unimi.it (F.T.); monica.bononi@unimi.it (M.B.)

* Correspondence: camilla.cattaneo@unimi.it (C.C.) Tel.: +39-0250319171

Supplementary Table S1. VIP coefficients of the models corresponding to each dependent variable

Variabile	Solvent odor	Honey odor	Caramel odor	Rum odor	Dried fig odor	Baked apple odor	Nutty odor	Solvent flavor	Honey flavor	Caramel flavor	Rum flavor	Dried fig flavor	Baked apple flavor	Nutty flavor
**ethyl acetate**	1,659	0,927	-0,97	-0,286	-0,847	-5,165	-1,54	4,495	4,932	-3,698	-2,613	1,051	8,126	0,731
**ethyl propionate**	-0,233	-0,159	0,13	0,098	0,077	0,805	0,187	-0,459	-0,847	0,623	0,468	-0,395	-1,252	-0,625
**2,4,5-trimethyl-1,3-dioxolane**	2,097	1,536	-1,06	-0,948	-0,505	-7,707	-1,833	4,865	8,151	-5,848	-4,478	2,584	11,499	0,93
**amyl alcohol + isoamyl alcohol**	4,721	1,978	-3,006	-0,432	-1,436	-12,027	-5,097	10,489	14,698	-19,427	-7,258	3,355	19,78	-7,741
**ethyl isobutyrate**	-0,311	-1,064	-0,217	1,352	-0,841	3,964	-0,075	0,369	-4,817	1,439	2,986	-2,684	-4,739	-1,767
**2,3 butanediol**	0,247	0,644	0,093	-0,769	0,463	-2,49	-0,057	0,112	2,978	-1,05	-1,812	1,371	3,011	0,175
**ethyl butyrate**	0,132	0,032	-0,089	0,066	-0,149	-0,296	-0,154	0,618	0,126	-0,036	-0,042	-0,265	0,499	-0,469
**ethyl 2-methyl butyrate**	-0,013	-0,383	-0,168	0,523	-0,385	1,331	-0,125	0,272	-1,605	0,139	1,029	-0,822	-1,422	-0,195
**ethyl isovalerate**	-0,495	-1,219	-0,148	1,446	-0,844	4,731	0,124	-0,225	-5,65	2,045	3,435	-2,677	-5,781	-0,698
**1-hexanol**	0,834	0,149	-0,635	0,267	-0,681	-1,485	-0,904	2,354	1,314	-2,179	-0,538	-0,028	2,899	-0,052
**isoamyl acetate**	1,824	0,342	-1,39	0,669	-1,78	-3,382	-1,895	5,718	2,304	-3,086	-0,918	-0,437	6,567	1,381
**limetol**	8,968	0,831	-7,245	4,2	-9,02	-13,465	-9,69	27,095	9,097	-18,455	-2,885	-2,455	28,91	5,734
**ethyl hexanoate**	11,644	-0,702	-10,226	7,756	-13,073	-11,236	-13,359	35,729	5,403	-26,152	0,557	-6,429	30,817	4,011
**linalool oxide**	0,512	0,134	-0,382	0,069	-0,34	-1,019	-0,511	1,126	1,072	-1,592	-0,499	0,412	1,935	0,792
**nonanal**	0,346	0,028	-0,289	0,139	-0,317	-0,477	-0,36	0,837	0,42	-0,942	-0,15	0,138	1,096	0,694
**phenylethyl alcohol**	5,362	0,982	-4,089	1,62	-4,262	-9,553	-5,772	14,6	8,761	-14,629	-3,643	0,44	18,699	0,708
**diethyl succinate**	10,939	5,647	-6,72	-1,386	-6,226	-32,252	-9,972	28,48	30,394	-23,805	-16,002	8,151	52,149	13,488
**alpha terpineol**	0,908	0,837	-0,375	-0,64	-0,049	-3,94	-0,738	2,039	4,223	-2,515	-2,387	1,407	5,603	0,297
**octanoic acid**	-0,811	0,553	0,949	-1,299	1,587	-0,903	1,087	-3,252	2,045	0,827	-1,548	1,829	-0,398	-0,466
**ethyl octanoate**	58,106	-31,793	-64,242	81,759	-104,227	38,039	-75,353	224,299	-111,21	-68,431	88,578	-112,496	56,163	30,74
**beta phenylethyl acetate**	0,722	0,188	-0,525	0,21	-0,692	-1,537	-0,726	2,336	1,042	-0,993	-0,46	-0,161	2,812	0,628
**alpha ionone**	0,33	-0,018	-0,286	0,23	-0,389	-0,337	-0,381	1,102	0,113	-0,613	0,037	-0,275	0,886	-0,013
**butyl octanoate**	-0,648	-0,334	0,407	0,099	0,368	1,891	0,566	-1,534	-1,816	1,453	0,964	-0,685	-3,103	-1,461
**ethyl decanoate**	22,522	-3,684	-20,897	18,974	-29,74	-14,326	-26,241	75,282	-3,503	-38,58	9,359	-20,742	52,264	14,617
**isoamyl octanoate**	-0,029	-0,016	0,02	0,011	0,014	0,082	0,018	-0,023	-0,09	0,08	0,051	-0,09	-0,145	-0,248
**ethyl dodecanoate**	0,379	-0,15	-0,41	0,345	-0,427	0,153	-0,455	0,663	-0,053	-1,627	0,196	0,163	0,505	0,996
